# Prognostic dynamic nomogram integrated with metabolic acidosis for in-hospital mortality and organ malperfusion in acute type B aortic dissection patients undergoing thoracic endovascular aortic repair

**DOI:** 10.1186/s12872-021-01932-8

**Published:** 2021-03-02

**Authors:** Jitao Liu, Weijie Liu, Wentao Ma, Lyufan Chen, Hong Liang, Ruixin Fan, Hongke Zeng, Qingshan Geng, Fan Yang, Jianfang Luo

**Affiliations:** 1 Department of Cardiology, Guangdong Cardiovascular Institute, Guangdong Provincial Key Laboratory of Coronary Heart Disease Prevention, Guangdong Provincial People’s Hospital, Guangdong Academy of Medical Sciences, Guangzhou, People’s Republic of China; 2 Center for Information Technology and Statistics, The First Affiliated Hospital of Sun Yat-Sen University, Guangzhou, People’s Republic of China; 3 School of Medicine, South China University of Technology, Guangzhou, People’s Republic of China; 4 Department of Cardiovascular Surgery, Guangdong Provincial Key Laboratory of South China Structural Heart Disease, Guangdong Provincial People’s Hospital, Guangdong Cardiovascular Institute, Guangdong Academy of Medical Sciences, Guangzhou, People’s Republic of China; 5 Department of Emergency and Critical Care Medicine, Guangdong Provincial People’s Hospital, Guangdong Academy of Medical Sciences, #96 Dongchuan Road, Yuexiu District, Guangzhou, 510080 Guangdong People’s Republic of China; 6 The Second School of Clinical Medicine, Southern Medical University, Guangzhou, People’s Republic of China

**Keywords:** Acute type B aortic dissection, Thoracic endovascular aortic repair, Nomogram, Base excess, Malperfusion

## Abstract

**Background:**

Organ malperfusion is a lethal complication in acute type B aortic dissection (ATBAD). The aim of present study is to develop a nomogram integrated with metabolic acidosis to predict in-hospital mortality and organ malperfusion in patients with ATBAD undergoing thoracic endovascular aortic repair (TEVAR).

**Methods:**

The nomogram was derived from a retrospectively study of 286 ATBAD patients who underwent TEVAR from 2010 to 2017 at a single medical center. Model performance was evaluated from discrimination and calibration capacities, as well as clinical effectiveness. The results were validated using a prospective study on 77 patients from 2018 to 2019 at the same center.

**Results:**

In the multivariate analysis of the derivation cohort, the independent predictors of in-hospital mortality and organ malperfusion identified were base excess, maximum aortic diameter ≥ 5.5 cm, renal dysfunction, D-dimer level ≥ 5.44 μg/mL and albumin amount ≤ 30 g/L. The penalized model was internally validated by bootstrapping and showed excellent discriminatory (bias-corrected c-statistic, 0.85) and calibration capacities (Hosmer–Lemeshow *P* value, 0.471; Brier Score, 0.072; Calibration intercept, − 0.02; Slope, 0.98). After being applied to the external validation cohort, the model yielded a c-statistic of 0.86 and Brier Score of 0.097. The model had high negative predictive values (0.93–0.94) and moderate positive predictive values (0.60–0.71) for in-hospital mortality and organ malperfusion in both cohorts.

**Conclusions:**

A predictive nomogram combined with base excess has been established that can be used to identify high risk ATBAD patients of developing in-hospital mortality or organ malperfusion when undergoing TEVAR.

**Supplementary Information:**

The online version contains supplementary material available at 10.1186/s12872-021-01932-8.

## Background

Acute type B aortic dissection (ATBAD) is an infrequent but lethal vascular disease. When it is complicated, thoracic endovascular aortic repair (TEVAR) is an effective treatment to prevent life-threatening complications like hemodynamic instability, severe organ malperfusion and even rupture of the aorta [1]. Despite prompt TEVAR, the mortality rate of these patients is still high (approximately 10% in the acute setting) [2], especially in patients with extensive tear range and multiple organ involvement [3–6].

Malperfusion caused by either a dynamic or static obstruction to the renal, mesenteric, spinal, or iliac arteries after TEVAR is devastating and demands emergency intervention if the end organs are threatened [6]. The early identification of risk factors for post-operative mortality and malperfusion, and then intervening reversible risk factors are essential to improve patients’ outcomes and limiting downstream costs. Several risk factors and predictive models for early prognosis have been presented [4, 7]. However, few studies considered metabolic acidosis, which is generally related to organ malperfusion and mortality [5]. Base excess (BE) is a pure indicator of metabolic acid–base balance disorders, regardless of respiratory condition [8, 9]. It was already shown that BE could be useful in predicting outcomes in critically ill patients after trauma or cardiac surgery, and patients with heart failure [9–11].

Therefore, the present study was designed to investigate the association of metabolic acidosis with adverse events, as well as to establish and validate a predictive nomogram integrated with metabolic acidosis for in-hospital mortality and organ malperfusion in patients with complicated ATBAD undergoing TEVAR.

## Methods

### Patients

In the derivation cohort, complicated ATBAD patients who underwent TEVAR procedures at our hospital from January 2010 to December 2017 were eligible for inclusion in this study. Patients with the following characteristics were excluded: (1) connective tissue disease, including Marfan and Loeys-Dietz syndromes; (2) blunt traumatic thoracic aortic injury; (3) malignant tumor; (4) previous aortic intervention; (5) pre-existing renal or visceral malperfusion; (6) incomplete data due to missing blood gas analyses. To examine the generalizability of the model, an external validation cohort of patients was prospectively collected separately, using the same inclusion and exclusion criteria as the derivation cohort in the same institution, from January 2018 to December 2019. Finally, the derivation and external validation cohort contained 286 and 77 patients, respectively. All patients underwent computed tomography (CT) scans with contrast enhanced, thin-sliced (range 0.75 to 1.25 mm) spiral CT (64-slice multidetector LightSpeed VCT; General Electric Fairfield, CT). Multiplanar reconstruction was performed by Aquarius iNtuition software (Terarecon, San Mateo, CA, USA). This study was approved by the ethics committee of the Guangdong Provincial People’s Hospital (#201807) and the need for informed consent was waived because of the retrospective nature of the analysis.

### Procedure

TEVAR was performed when ATBAD was complicated [1]. The details of the procedure at our hospital had been previously described [12]. Briefly, the procedures were performed with suitable anatomy in a cardiac catheterization room under local anaesthesia. All stent grafts were deployed retrogradely via percutaneous femoral artery access to obliterate the proximal entry tear. The left subclavian artery (LSA) were covered when necessary to obtain 1.5–2 cm proximal landing zone. The choice of reconstruction of the LSA mainly depends on the vertebrobasilar circulation by operators. The diameters of aortic stent grafts were generally oversized by 5% to 10% according to the aortic pathologies. Most patients were treated with a single endograft prosthesis, and additional pieces were placed only when the initial graft did not produce the desired result of coverage of the entry tear and expansion of the true lumen as determined by angiography but the distal end of the stent-graft was still above the diaphragm. No branched stent-graft was used during the study period and balloon angioplasty of the proximal seal zone was avoided if possible to prevent retrograde extension of the dissection into the aortic arch.

### Data collection and definitions

Retrospective data on age, sex, medical history, coexisting medical conditions, imaging features, operation parameters, and follow-up records were collected and analyzed. In our center, the blood gas analyses were performed at admission, in the each morning and at the time of disease progression. Each patient had at least one measurement before the procedure. The lowest pH, the lowest bicarbonate concentration, the nadir BE and the highest lactate were employed.

ATBAD was defined as a type B aortic dissection occurring less than 14 days after the onset of symptoms [1]. Complicated type B aortic dissection was described as persistent or recurrent pain, uncontrolled hypertension despite full medication, early aortic expansion, malperfusion, and signs of rupture (haemothorax, increasing periaortic and mediastinal haematoma) [1]. Patients with renal dysfunction were those with an estimated glomerular filtration rate (eGFR) lower than 60 ml/min/1.73m^2^ at admission [13]. The maximum pre-operative outer to outer aortic diameter was measured orthogonal to the vessel centreline. The supply of the abdominal arterises (coeliac artery, superior mesenteric artery, left renal artery and right renal artery) were assessed by multiplanar reconstructed enchanced-CT. The diagnosis of malperfusion in the context of ATBAD was based on the patient’s presenting symptoms in addition to CT confirmation and intra-operative visualization of obstruction to any aortic branch vessels [6, 14].

The endpoint of interest was described as a composite outcome of in-hospital death or new-onset organ malperfusion including visceral malperfusion, renal malperfusion, lower extremity malperfusion and spinal cord malperfusion according to the description of White et al. [14]. A patient having multiple events was considered as having only one event.

### Statistical analysis

Continuous data were presented as mean ± standard deviation or median (quartiles 1 to 3) and were compared using the Student's t-or the Mann–Whitney U tests depending on distribution. The Shapiro–Wilk test was selected for the normality test. Qualitative data are presented as frequencies (percentages) and compared using the Chi-square or Fisher’s exact tests.

To establish the predictive model, the least absolute shrinkage and selection operator regression (LASSO) was used to select variables in the predictive model. By applying multivariate logistic regression, we established a predictive model. An optimal penalization factor was determined using the “pentrace” function in Harrell’s R package “rms” [15] to avoid overfitting. The bootstrapping approach was used for internal model validation as it is considered more efficient than split-data and cross-validation methodologies [15]. Bootstrapping replicates the process of sample generation from an underlying population by drawing samples with replacement from the original data set. The model can be constructed and validated with 100% of the number of subjects using bootstrapping, which make the prediction and internal validity accurate and stable, especially when the sample size is small. Nevertheless, cross-validation or split-data uses part of subjects for model construction and the other for validation, which might result in unstable and biased estimates of performance. The risk predictive model for in-hospital mortality and organ malperfusion in ATBAD patients undergoing TEVAR was presented using a nomogram. In addition, a web-based dynamic prediction tool based on the nomogram has been created to facilitate the calculation and aid in the decision-making process in clinical practice (https://sycardiovascular.shinyapps.io/DynNomappBE_version2/).

Model performance was assessed under three aspects: (1) The discriminatory capacity was evaluated by the area under the receiver operating characteristics (ROC) curve (AUC), while the bias-corrected AUC was calculated using bootstrapping 1000 times. (2) The calibration ability was evaluated using the following four different methods: the Hosmer–Lemeshow test; calibration plot; the Brier score, as well as the intercept and slope of the calibration. (3) The clinical effectiveness was assessed using the decision curve analysis (DCA). In addition, we also derived an optimal cut-off threshold to determine the positive predictive value (PPV) and the negative predictive value (NPV) to assess clinical usefulness. A sensitivity analysis was also performed in the entire cohort.

All tests were two-tailed and a *P* values of < 0.05 were considered statistically significant. The optimal cut-off value was determined by the ROC curve according to the Youden index. All statistical analyses were performed using R software (version 3.5.1).

## Results

### Cohorts characteristics

A total of 363 complicated ATBAD patients who underwent TEVAR were enrolled in this study, with 286 of them in the derivation cohort and 77 in the external validation cohort. The majority of the participants were male (n = 326, 89.8%) in both cohorts and median age was 52 years (IQR: 45–62) (Table 1). The derivation and validation cohorts showed relatively well balanced features, except for a higher eGFR level and a lower incidence of maximum aortic diameter ≥ 5.5 cm in the derivation cohort (Table 1). The prevalence of in-hospital mortality or organ malperfusion was similar between the derivation and external validation cohorts (n = 41 [14.3%] vs n = 11 [14.3%], *P* = 0.991, Table 2).

**Table 1:** Baseline characteristics of the study population

Variables	Derivation cohort (n = 286)	External validation cohort (n = 77)	*P*
Age, years	53 ± 11	55 ± 12	0.276
Male	255 (89.2)	71 (92.2)	0.433
Comorbidities			
Hypertension	246 (86.0)	64 (83.1)	0.523
Diabetes mellitus	15 (5.2)	6 (7.8)	0.411
Coronary artery disease	37 (12.9)	14 (18.2)	0.240
Dyslipidemia/statin use	33 (11.5)	11 (14.3)	0.512
Renal dysfunction	54 (18.9)	15 (19.5)	0.905
Stroke	11 (3.8)	6 (7.8)	0.218
Anemia	140 (49.0)	35 (45.5)	0.586
Organ malperfusion			
Extremity	31 (10.8)	10 (13.0)	0.597
Abdominal organs	175 (61.2)	45 (58.4)	0.661
Laboratory findings			
Base excess value	− 2.6 (− 5.7 to − 0.1)	− 2.0 (− 4.9 to 0.3)	0.257
Base excess stratification			0.682
≥ 0	68 (23.8)	20 (26.0)	
− 5 to 0	138 (48.3)	41 (53.2)	
− 10 to − 5	61 (21.3)	12 (15.6)	
≤ − 10	19 (6.6)	4 (5.2)	
pH	7.40 (7.36–7.44)	7.40 (7.36–7.43)	0.623
Bicarbonate, mmol/L	23.1 (21.0–24.5)	23.3 (21.6–25.2)	0.470
Lactate	1.3 (0.8–2.3)	1.3 (0.9–2.2)	0.344
Total cholesterol, mmol/L	4.4 ± 0.9	4.3 ± 1.1	0.311
Triglyceride, mmol/L	1.4 (1.0–1.9)	1.3 (1.0–1.9)	0.659
LDL-c, mmol/L	2.6 (2.1–3.1)	2.8 (1.9–3.2)	0.754
Albumin ≤ 30 g/L	101 (35.3)	19 (24.7)	0.078
D-dimer ≥ 5.44 μg/mL	63 (22.0)	24 (31.2)	0.095
eGFR, mL·min^−1^·1.73 m^−2^	77.1 (46.3–96.9)	56.1 (42.5–79.9)	0.002
Imaging findings			
Diameter ≥ 55 mm	22 (7.7)	13 (16.9)	0.015
Extent of dissection			0.596
Confined in thoracic aorta	52 (18.2%)	12 (15.6%)	
Extended to abdominal aorta	234 (81.8%)	65 (84.4%)	
False lumen patency			0.278
Patent	200 (69.9)	60 (77.9)	
Partially thrombosed	81 (28.3)	15 (19.5)	
Completely thrombosed	5 (1.7)	2 (2.6)	
Ejection fraction, %	64.5 (62.0–68.0)	65.0 (62.0–69.0)	0.956
Blood supply of abdominal arteries			
Coeliac artery (TL/FL/TF)	185 (64.7)/92 (32.2)/9 (3.1)	47 (61.0)/25 (32.5)/5 (6.5)	0.372
Superior mesenteric artery (TL/FL/TF)	217 (75.9)/57 (19.9)/12 (4.2)	60 (77.9)/12 (15.6)/5 (6.5)	0.499
Left renal artery (TL/FL/TF)	196 (68.5)/81 (28.3)/9 (3.1)	52 (67.5)/22 (28.6)/3 (3.9)	0.908
Right renal artery (TL/FL/TF)	209 (73.1)/68 (23.8)/9 (3.1)	54 (70.1)/18 (23.4)/5 (6.5)	0.397

Data are presented as n (%) or mean ± standard deviation or median (quartiles 1 to 3); eGFR, estimated glomerular filtration rate; LDL-c, Low-density lipoprotein cholesterol; TL, true lumen; FL, false lumen; TF, true lumen and false lumen

**Table 2:** In-hospital outcomes

Variables	Derivation cohort	External validation cohort	*P*
Mortality	20 (7.0)	6 (7.8)	0.809
Organ malperfusion	25 (8.7)	7 (9.1)	0.923
Lower extremity malperfusion	7 (2.4)	2 (2.6)	0.940
Visceral malperfusion	4 (1.4)	2 (2.6)	0.611
Renal malperfusion	14 (4.9)	3 (3.9)	0.713
Spinal cord malperfusion	5 (1.7)	2 (2.6)	0.631
Hospital stay, days	17.0 (13.0–23.0)	17.0 (13.0–21.0)	0.591
Intensive care unit stay, hours	72.0 (18.0–144.0)	52.0 (4.0–133.5)	0.927

### Model construction

After the initial selection and the elimination of redundant candidates based on LASSO regression analysis, the final selection of predictors was performed by multivariate logistic regression analysis (Additional file 1: Table S1). To enhance the clinical use, the continuous BE value was divided into four groups (≥ 0; − 5 to 0; − 10 to − 5; ≤ − 10) [5] and enrolled into multivariate analysis (Additional file 1: Table S2). As a result of no significant difference between − 5 to 0 group and ≥ 0 group, these two groups were combined to form the low risk group (> − 5). Therefore, BE was split into three groups: low risk group (> − 5); moderate risk group (− 10 to − 5) and high risk group (≤ − 10). Multivariate analysis demonstrated that moderate and high risk BE group, maximum aortic diameter ≥ 55 mm, renal dysfunction and D-dimer ≥ 5.44 μg/mL were independent risk factors for in-hospital mortality and organ malperfusion in complicated ATBAD patients undergoing TEVAR (Table 3). Each predictor received a score based on the regression coefficient derived from the multivariate logistic regression model and summed to the final risk prediction model.

**Table 3:** Multivariable predictors of in-hospital mortality and organ malperfusion (derivation cohort)

Variables	Multivariate		
	OR	95% CI	*P* value
Base excess*			< 0.001
Moderate risk versus low risk	4.98	2.01–12.32	0.001
High risk versus low risk	8.40	2.09–33.84	0.003
Renal dysfunction	3.99	1.62–9.81	0.003
D-dimer ≥ 5.44 μg/mL	3.82	1.55–9.46	0.004
Albumin ≤ 30 g/L	4.51	1.88–10.80	0.001
Diameter ≥ 55 mm	6.51	1.89–22.45	0.003

The predictive model was adjusted by pH, lactate, bicarbonate, decreased peripheral arterial pulse, hypertension, coronary artery disease, stroke, anemia, estimated glomerular filtration rate < 60 mL/min/1.73 m^2^, extent of the dissection, blood supply of visceral arteries (coeliac artery, superior mesenteric artery, left renal artery and right renal artery)

OR, odds ratio; CI, confidence interval

*Base excess was divided into low risk group (> − 5), moderate risk group (− 10 to − 5) and high risk group (≤ − 10)

### Internal validation

**Fig. 1:**
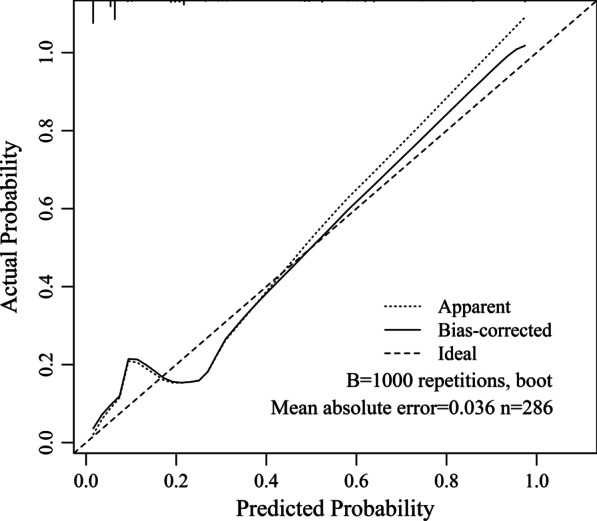
Internal calibration plot for the predictive nomogram by bootstrapping in the derivation cohort

**Fig. 2:**
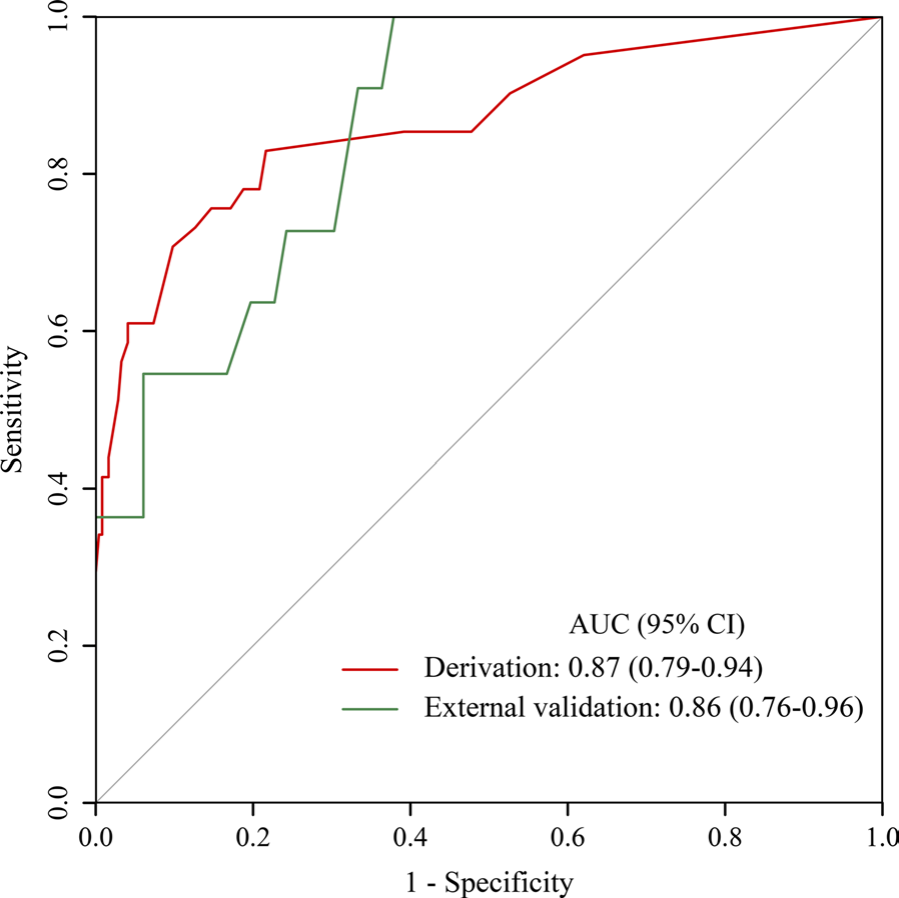
Receiver operating characteristic (ROC) curve to assess the discrimination performance of the model

**Fig. 3:**
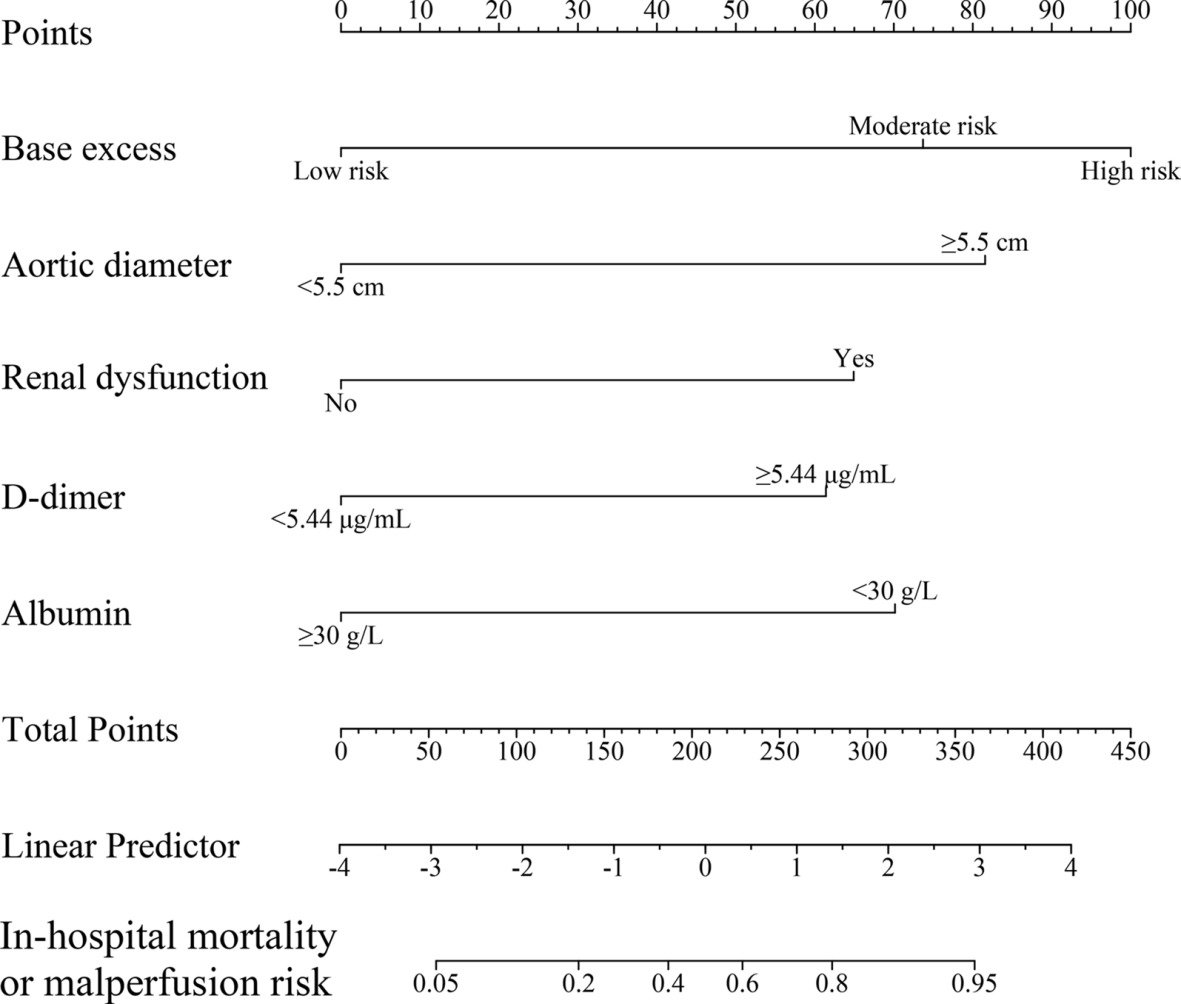
Penalized-nomogram for the in-hospital mortality and organ malperfusion risk in ATBAD patients undergoing TEVAR. To estimate the probability of the occurrence of in-hospital mortality or organ malperfusion for a given patient, add up the points identified on the point scale for each variable

A Brier score of 0.072 and Hosmer–Lemeshow goodness-of-fit tests with 5.59 of chi-square value (*P* = 0.471) in the derivation cohort suggest a good fitting of the model. To further detect any deviation between observed and predicted events, we internally validated the model by bootstrapping (1000 iterations) the slope and intercept of the calibration plot and the AUC. The original AUC was 0.87, while the bias-corrected estimate was 0.85. The original intercept and slope of the calibration plot were 0 and 1, respectively. The bias-corrected values were -0.13 and 0.89 indicating a mild overfitting. Therefore, it was added a penalty using the “pentrace” function in Harrell’s R package “rms” [15] to improve the model fit and obtain a new calibration plot (Fig. 1). After applying the penalty factor, the original AUC was 0.87 (Fig. 2) and the bias-corrected AUC was 0.85, while the bias-corrected estimates of the intercept and slope were − 0.02 and 0.98 (Fig. 1), respectively. Based on these results, a nomogram was configured (Fig. 3).

### External validation

The external validation of the predictive model was performed with data prospectively collected separately from January 2018 to December 2019 at the same institute from which the previous data were collected. Of the 77 complicated ATBAD patients who underwent TEVAR, 11 developed in-hospital mortality or organ malperfusion. The validation AUC was 0.86 (Figs. 2), which was consistent with the derivation cohort AUC of 0.87 (*P* = 0.927). The Brier score was 0.097, indicating good model calibration. It was also performed a sensitivity analysis on patients from both cohorts and the predictive model continued to perform well to predict the incidence of in-hospital mortality and organ malperfusion (AUC, 0.86; 95% confidence interval [CI] 0.80–0.92).

### Clinical effectiveness

**Fig. 4:**
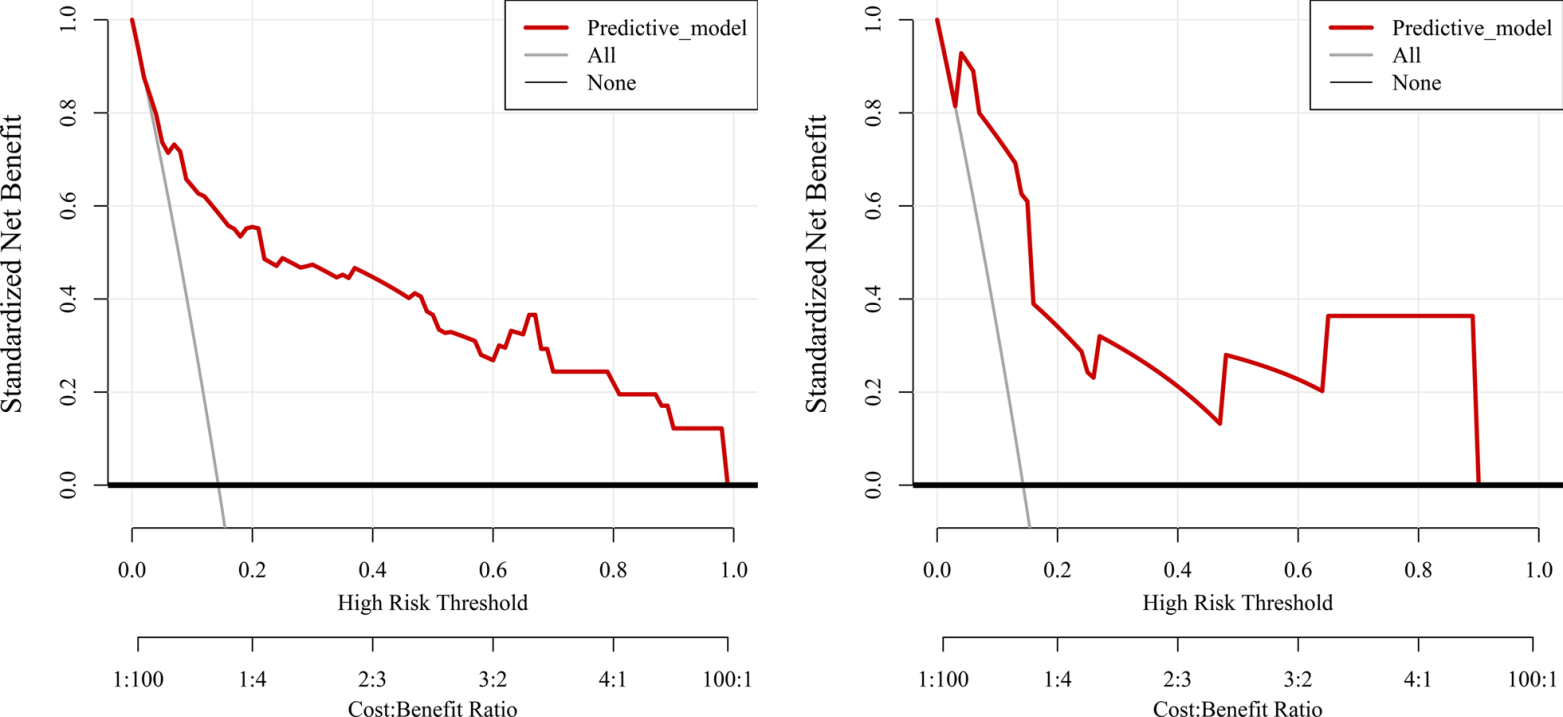
Decision curve analysis (DCA) for the predictive nomogram in the derivation (**a**) and external validation (**b**) cohorts. Black line: All patients died; Grey line: None of the patients died; Red line: the predictive nomogram model

DCA was used to assess the clinical effectiveness of the predictive model. The net benefits of the risk model were obviously more prominent than “treating-all-patients” or “treating-none”, in the derivation (Fig. 4a) and external validation cohorts (Fig. 4b). In addition, we derived the optimal cut-off threshold for the predictive model providing PPV and NPV that might provide clinically useful information. An optimal model threshold score (0.39) gave an NPV of 0.94 and a PPV 0.71 in the derivation cohort. The threshold was carried forward and the NPV remained high (0.93), whereas the PPV decreased to 0.60 in the external validation cohort.

## Discussion

In a cohort of 363 complicated ATBAD patients who underwent TEVAR, five risk factors for the occurrence of in-hospital mortality or organ malperfusion were identified from the multivariate analysis of our derivation cohort data: perioperative nadir BE, aortic diameter ≥ 5.5 cm, renal dysfunction, D-dimer level ≥ 5.44 μg/mL and albumin amount ≤ 30 g/L. The nomogram incorporated easily accessible clinical, imaging and laboratory data and showed excellent capacity of discrimination, calibration and clinical effectiveness, thus making it a clinically valuable tool.

The first and new risk factor identified in our patients was the perioperative nadir BE. Metabolic acidosis is closely associated with the dysfunction of several organs and systems and with increased mortality [5]. It is characterized by a reduction in serum bicarbonate concentration, BE and, consequently, acidification of blood pH. BE is a pure indicator of metabolic acid–base balance disorders, regardless of respiratory condition, while pH and serum bicarbonate concentration are variable due to respiratory compensation, pre-hospital care and physical conditions [9]. As expected, it was found that pH or bicarbonate were significantly risk factors for in-hospital mortality and new-onset organ malperfusion after TEVAR in the univariate analysis (*P* < 0.001 for both). However, their effect were attenuated after adjusting the BE in the derivation set (*P* = 0.081 and 0.213, respectively), reflecting its predictive superiority.

Multiple studies have demonstrated factors associated with organ malperfusion and in-hospital mortality in ATBAD patients undergoing TEVAR [3, 4, 6]. For instrance, branch vessel involvement was reported to be an independent risk factor for in-hospital mortality, which emphasized the importance of early identification and intervention in organ malperfusion [4]. The present study assessed the association between in-hospital mortality and organ malperfusion with BE in ATBAD patients undergoing TEVAR, demonstrating lower BE was related to increasing events (*P* < 0.001). In addition, compare to malperfusion, which is obscure and rely on symptoms, physical examination, or imaging findings, the acidosis is a directly quantitative measurement of ischemia and organ injury.

According with previous studies [4, 7, 16], patients with a maximum aortic diameter ≥ 5.5 cm, renal dysfunction or albumin amount ≤ 30 g/L were more likely to experience in-hospital mortality or organ malperfusion. D-dimer level ≥ 5.44 μg/mL was the last identified risk factor. This cutoff value (5.44 μg/mL) is slightly lower than that found in a previous study by Dan and colleagues [17]. These authors found that a D-dimer level ≥ 5.67 μg/mL provided the most optimal sensitivity and specificity in predicting in-hospital mortality. This discrepancy might be explained by the type of aortic dissection, the false lumen status and features of lesion in the dissected aorta [18].

Identifying a patient that whether he or she would suffer from in-hospital mortality or organ malperfusion is an important step in the process of decision making before intervention. This predictive model may be helpful in determining treatment strategies. Selective branched stent-graft combined or not with flap fenestration [3, 6, 19, 20] or use of the composite device (proximal stent grafts and distal bare aortic stent) [21] might be a optimal choice for those susceptiable to organ malperfusion to maximize the organ perfusion restoration. Cerebrospinal-fluid (CSF) drainage, in addition to medical theraphy, for reduction of CSF pressure was recommended for prevention and treatment of spinal cord injury as part of conservative approach or bridge to surgery [22]. Furthermore, hemodialysis might be beneficial to correct metabolic acidosis and reduce the risk of developing severe organ malperfusion after TEVAR.

In addition, the clinical utility of the model was further demonstrated by it’s high NPV, which means that clinicians may be more confident in excluding patients with low probability of suffer from in-hospital mortality or organ malperfusion. Another attractive aspect of our nomogram-model is its clinical applicability, as it can be employed at the bedside. The risk probability of in-hospital mortality or organ malperfusion in ATBAD patients after TEVAR can be scaled by matching the total points for a given patient. For instance, a hypothetical hospitalized ATBAD patient with BE = -8, maximum aortic diameter = 5.8 cm, D-dimer = 6.2 μg/mL and albumin = 28 g/L has a total score > 280 that corresponds to a prediction of approximately 80% risk of suffer from in-hospital mortality or malperfusion. Furthermore, a web-based calculator can be used to increase the approachability of the predictive model. This nomogram tool is relatively simple to understand and can improve communication between patients and clinicians.

There are several limitations deserved to be noted. First, this study is a retrospective project performed with hospital data from a single medical center. Secondly, we did not analyze the effects of the administration of drugs that can correct the acid–base imbalance, such as sodium bicarbonate. However, lacking of this data would only underestimate the predictive capacity of the model. The inclusion of this analysis would not significantly change the prediction model profile and would probably only reinforce the data presented here. Thirdly, data prospectively collected at the same center was used for external validation in the present study, which restricted the generalizability of this predictive model to some extent. A larger prospective study and external validation studies at other centers are needed to determine the accuracy of this risk prediction model.

## Conclusions

BE is a important risk factor of complicated ATBAD patients undergoing TEVAR. A effective predictive nomogram for early identification of high risk patients to develop in-hospital mortality or organ malperfusion after TEVAR has been established, thus allowing clinicians to better customize ATBAD management to the individual and provide prompt and effective interventions.

## Supplementary Information

**Additional file 1: Table S1**. Multivariable predictors of in-hospital mortality and organ malperfusion (derivation cohort). **Table S2**. Multivariable predictors of in-hospital mortality and organ malperfusion (derivation cohort).

## Data Availability

The datasets used and/or analyzed during the current study are de-identified and available from the corresponding author on reasonable request.

## Abbreviations

ATBAD: Acute type B aortic dissection (ATBAD)
TEVAR: Thoracic endovascular aortic repair
BE: Base excess
LSA: Left subclavian artery
DCA: Decision curve analysis
PPV: Positive predictive value
NPV: Negative predictive value
CSF: Cerebrospinal-fluid
