# Supplementary material for: Prognostic dynamic nomogram integrated with metabolic acidosis for in-hospital mortality and organ malperfusion in acute type B aortic dissection patients undergoing thoracic endovascular aortic repair
Source: BMC Cardiovasc Disord. 2021 Mar 2;21:120. doi: 10.1186/s12872-021-01932-8 (PMC7927380; doi:10.1186/s12872-021-01932-8)
Supplement: Supplementary file 1 — Additional file 1: Table S1. Multivariable predictors of in-hospital mortality and organ malperfusion (derivation cohort). Table S2. Multivariable predictors of in-hospital mortality and organ malperfusion (derivation cohort). [file 12872_2021_1932_MOESM1_ESM.docx]

**Online Supplementary material**

**Supplementary Table S1.** **Multivariable predictors of in-hospital mortality and organ malperfusion (derivation cohort)**

| variables | Multivariate | | |
| --- | --- | --- | --- |
|  | OR | 95% CI | *P* value |
| Base excess | 0.86 | 0.78 to 0.94 | 0.002 |
| Renal dysfunction | 3.69 | 1.50 to 9.07 | 0.004 |
| D-dimer≥5.44 μg/mL | 3.41 | 1.40 to 8.31 | 0.007 |
| Albumin≤30 g/L | 4.14 | 1.73 to 9.88 | 0.001 |
| Diameter≥55 mm | 7.18 | 2.17 to 23.71 | 0.001 |

The predictive model was adjusted by pH, lactate, bicarbonate, decreased peripheral arterial pulse, hypertension, coronary artery disease, stroke, anemia, estimated glomerular filtration rate <60 mL/min/1.73 m^2^ , extent of the dissection, blood supply of abdominal arteries (coeliac artery, superior mesenteric artery, left renal artery and right renal artery). *OR*, Odds ratio; *CI*, confidence interval.

**Supplementary Table S2.** **Multivariable predictors of in-hospital mortality and organ malperfusion (derivation cohort)**

| variables | Multivariate | | |
| --- | --- | --- | --- |
|  | OR | 95% CI | *P* value |
| Base excess |  |  | 0.001 |
| -5 to 0 vs ≥0 | 0.66 | 0.17 to 2.50 | 0.538 |
| -10 to -5 vs ≥0 | 3.74 | 1.07 to 13.08 | 0.039 |
| ≤-10 vs ≥0 | 6.26 | 1.19 to 32.93 | 0.031 |
| Renal dysfunction | 4.15 | 1.66 to 10.34 | 0.002 |
| D-dimer≥5.44 μg/mL | 3.96 | 1.59 to 9.90 | 0.003 |
| Albumin≤30 g/L | 4.52 | 1.88 to 10.83 | 0.001 |
| Diameter≥55 mm | 6.45 | 1.87 to 22.18 | 0.003 |

The predictive model was adjusted by pH, lactate, bicarbonate, decreased peripheral arterial pulse, hypertension, coronary artery disease, stroke, anemia, estimated glomerular filtration rate <60 mL/min/1.73 m^2^ , extent of the dissection, blood supply of abdominal arteries (coeliac artery, superior mesenteric artery, left renal artery and right renal artery). *OR*, Odds ratio; *CI*, confidence interval.
